# Sea of Majorana fermions from pseudo-scalar superconducting order in three dimensional Dirac materials

**DOI:** 10.1038/s41598-017-07298-2

**Published:** 2017-08-15

**Authors:** Morteza Salehi, S. A. Jafari

**Affiliations:** 1 Department of Physics, Sharif University of Technology, Tehran, 11155-9161 Iran; 2 Theoretische Physik, Universität Duisburg-Essen, 47048 Duisburg, Germany

## Abstract

We suggest that *spin*-*singlet pseudo*-*scalar s*-*wave* superconducting pairing creates a two dimensional sea of Majorana fermions on the surface of three dimensional Dirac superconductors (3DDS). This pseudo-scalar superconducting order parameter Δ_5_, in competition with scalar Dirac mass *m*, leads to a topological phase transition due to band inversion. We find that a perfect Andreev-Klein reflection is guaranteed by presence of *anomalous Andreev reflection* along with the conventional one. This effect manifests itself in a resonant peak of the differential conductance. Furthermore, Josephson current of the Δ_5_|*m*|Δ_5_ junction in the presence of anomalous Andreev reflection is fractional with 4*π* period. Our finding suggests another search area for condensed matter realization of Majorana fermions which are beyond the vortex-core of p-wave superconductors. The required Δ_5_ pairing can be extrinsically induced by a conventional s-wave superconductor into a three dimensional Dirac material (3DDM).

## Introduction

Band topology in insulators and superconductors is connected with the change in the sign of the gap parameter which in turn creates zero energy states at the location of gap kink. This mechanism in the case of insulators leads to gapless surface modes protected by a topological invariant^1–4^. When the spectral gap is of the superconducting (pairing) nature, the sign change of the oder parameter gives rise to midgap states^5, 6^. These topologically protected modes will be Majorana zero modes, which are their own anti-particles^7–10^. To realize Majorana fermions (MFs) various scenarios have been proposed which involve closing and re-opening the superconducting gap in one way or another. Gaping chiral modes of topological insulators (TIs) by Zeeman field and superconducting pairing gives rise to MFs in the interface region where the strength of these two gapping mechanisms are equal^11, 12^. In one-dimensional nano-wires this can be achieved by the competition between a polarizing Zeeman field, and depolarizing spin-orbit interaction^13, 14^. In two-dimensions, the vortex core of a p-wave SC binds a MF^15^. The required p-wave SC can be engineered on the surface of a TI by proximity to a conventional s-wave SC^11^. The above scenarios are: (i) limited to low dimensions, (ii) require a TR breaking by a Zeeman field, and (iii) require p-wave superconductors which are not abundant in nature.

**Figure 1:**
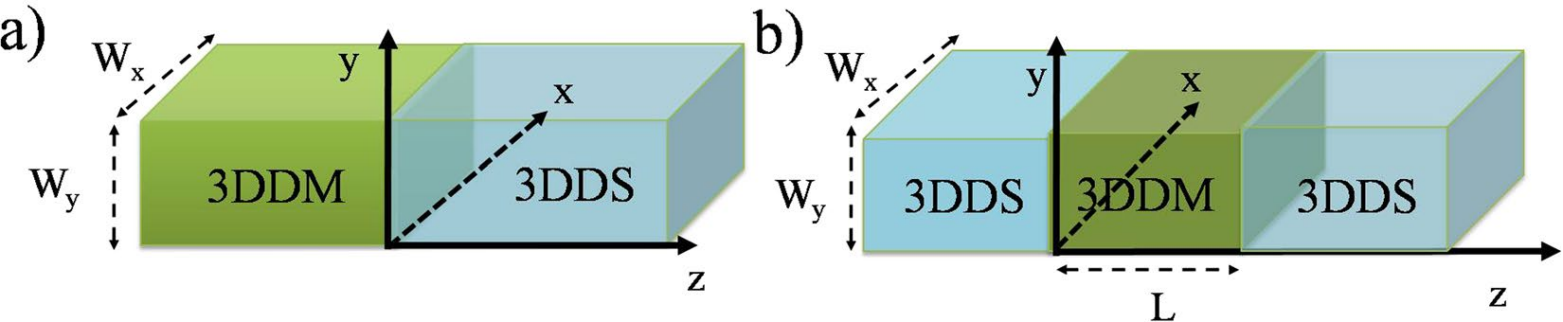
(**a**) Schematic illustration of 3DDM|3DDS junction (*m*|Δ_5_). We assume a 3DDM can be superconductor by proximity effect. (**b**) The Josephson junction of 3DDS|3DDM|3DDS (Δ_5_|*m*|Δ_5_).

In this report we propose yet another *three dimensional* system that admits a two dimensional sheet of MFs without requiring a Zeeman field. As such the resulting MFs are not associated with a vortex core and hence no low-energy excitations other than MFs exist^16, 17^. We find that a peculiar *pseudo*-*scalar* superconducting order parameter, Δ_5_ can give rise to a two-dimensional sheet of MFs when it is interfaced with a 3DDM. To set the stages for our finding, let us start by noting that in a one-band situation described by a parabolic band dispersion, the strength of the gap is characterized by a (scalar) gap parameter. However in 3DDM where the relevant degrees of freedom are described by the Dirac equation^18^, new opportunities can arise. The low-energy effective Hamiltonian of 3DDM candidate compounds like Cu_*x*_(Bi_2_Se_3_)_1−*x*_^19, 20^, Na_3_Bi_1−*x*_Sb_*x*_^21, 22^ and Cd_3_(As_1−*x*_P_*x*_)_2_^21, 22^ are described by a Dirac equation in the Γ point of their Brillouin zone. The material Cu_*x*_Bi_2_Se_3_ is a Dirac superconductor below 3.8 K^23^ and hence is a 3DDS. For such a 3DDM, the low-energy degrees of freedom are described by 4-component Dirac spinor. The most general superconducting pairing is $\bar{\psi }\hat{{\rm{\Delta }}}{\psi }_{c}={\psi }^{\dagger }{\gamma }^{0}\hat{{\rm{\Delta }}}{\psi }_{c}$ where *ψ*_*c*_ satisfies the same Dirac equation as *ψ*, but with opposite charge and is covariant under Lorentz transformation. The 4 × 4 pairing matrix $\hat{{\rm{\Delta }}}$ can be expanded in terms of a basis composed of **1**, four *γ* ^*μ*^, *γ* ^5^, four *γ* ^5^ *γ* ^*μ*^ and six anti-commutators *σ* ^*μν*^ = *i*[*γ* ^*μ*^, *γ* ^*ν*^]/2^24^ as $\hat{{\rm{\Delta }}}={{\rm{\Delta }}}_{s}{\bf{1}}+{{\rm{\Delta }}}_{\mu }{\gamma }^{\mu }+{{\rm{\Delta }}}_{5}{\gamma }^{5}+{{\rm{\Delta }}}_{5\mu }{\gamma }^{5}{\gamma }^{\mu }+{{\rm{\Delta }}}_{\mu \nu }{\sigma }^{\mu \nu }$. Then the pairing Δ_*s*_ will be the conventional scalar pairing, while Δ_5_ will be a pseudo-scalar pairing under the Lorentz transformation. Similarly Δ_*μ*_, Δ_5*μ*_, Δ_*μν*_, will be vector, pseudo-vector and tensor superconducting pairings^25^. We find that only the pseudo-scalar superconducting gap Δ_5_ competes with scalar Dirac gap *mγ* ^0^ and leads to gap closing. Therefore placing a *m* dominated region (*i*.*e*. a 3DDM) next to a Δ_5_ dominated region denoted as *m*|Δ_5_ junction (Fig. 1), gives rise to a two-dimensional sheet of Majorana fermions. This mechanism, *does not require p*-*wave pairing*, *nor a magnet to break time*-*reversal*. Instead, it requires a peculiar form of pseudo-scalar superconducting order parameter that changes sign in the mirror. We then proceed to show that such a system belongs to the DIII topological class allowing *Z*-number classification which in turn guarantees the existence of two-dimensional Majorana sea (2DMS) on the region where the strength of *m* and Δ_5_ are equal. We further corroborate our results by showing an anomalous Andreev reflection which couples electron and holes with the same spin direction, genuinely creates a perfect Andreev-Klein effect independent of angle of incidence. This effect manifests itself in the robust zero-mode resonance peak in the differential conductance and fractional supercurrent in the Δ_5_|*m*|Δ_5_ Josephson junction.

## Results

### Gap closing and topological phase transition

We consider a Dirac material with a single Dirac cone,

1 $$ {\mathcal H} ({\bf{k}})={v}_{F}{\gamma }_{0}\,(\hslash \gamma .{\bf{k}}+m{v}_{F}).$$

**Figure 2:**
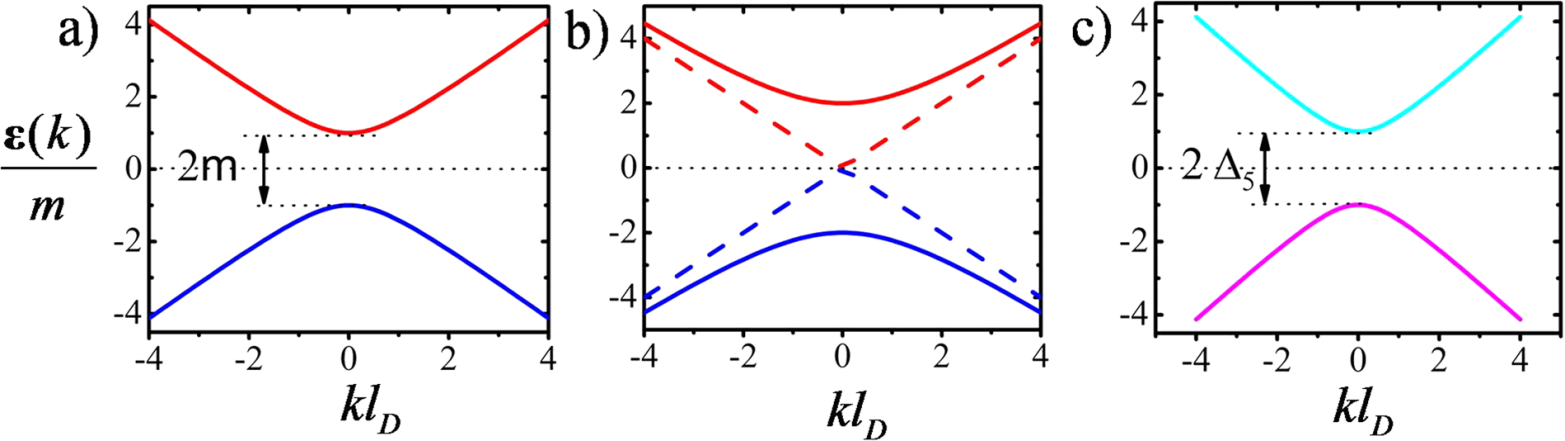
(**a**) Dispersion relation of 3DDM regime. (**b**) Gap closing and topological phase transition for one band whereas the other one remains trivial. (**c**) Dispersion relation of 3DDS regime. In these results we set *E*_*F*_ = 0. The length scale *l*_*D*_ = *ħ*/*mv*_*F*_ is set by the energy gap of the 3DDM.

We use the representation ${\gamma }^{0}={\tau }_{3}\otimes {\sigma }_{0}$ and ${\gamma }^{j}={\tau }_{1}\otimes i{\sigma }_{j}$ for the Clifford algebra where *σ*_*j*_ and *τ*_*j*_ are Pauli matrices acting on spin and band spaces, respectively. Also, *σ*_0_ and *τ*_0_ are the 2 × 2 unit matrices. The **k** is the wave vector of excitations. In four space-time dimensions one can also construct *γ* ^5^ = *iγ* ^0^ *γ* ^1^ *γ* ^2^ *γ* ^3^ which will be very essential for our discussion in this paper. In Eq. (1), the mass term is of the ordinary *mγ* ^0^ nature and is responsible for the band gap, and *v*_*F*_ is the Fermi velocity. The pairing Hamiltonian for such a system is:

2 $${H}_{BCS}=\frac{1}{2}\int \,d{\bf{r}}\,({\psi }^{\dagger }\quad {\psi }_{c}^{\dagger })\,{H}_{{\rm{DBdG}}}\,(\begin{array}{c}\psi \\ {\psi }_{c}\end{array}),$$

corresponding to which the Dirac-Bogoliubov-deGennes (DBdG) equation in **k**-space is,

3 $$(\begin{array}{cc}{\mathscr{H}}({\bf{k}})-{E}_{F} & {\gamma }_{0}\hat{{\rm{\Delta }}}{e}^{i\varphi }\\ {\hat{{\rm{\Delta }}}}^{\dagger }{\gamma }_{0}{e}^{-i\varphi } & {E}_{F}+{\mathscr{C}}\,{\mathscr{H}}\,({\bf{k}})\,{{\mathscr{C}}}^{-1}\end{array})\,(\begin{array}{c}u\\ v\end{array})=\varepsilon \,(\begin{array}{c}u\\ v\end{array}),$$

where *ε* is the energy of eigenstate with respect to the chemical potential *E*_*F*_. Here *ϕ* is the macroscopic phase of superconductor, *u*(*v*) is the electron (hole) part of BdG wave function in Nambu space. The anti-unitary operator ${\mathscr{C}}={\gamma }_{2}{\gamma }_{0}K$ is the charge-conjugation of Eq. (1), where *K* is the complex-conjugation: This means that Lorentz transformation for ${\bar{\psi }}_{c}={\psi }_{c}^{\dagger }{\gamma }^{0}$ is the inverse of the Lorentz transformation for *ψ*. Therefore requiring the superconducting pairing to be Lorentz invariant^24, 26^, the scalar (conventional) superconducting pairing is given by ${\bar{\psi }}_{c}{{\rm{\Delta }}}_{s}{\bf{1}}\psi ={\psi }_{c}^{\dagger }{\gamma }^{0}{{\rm{\Delta }}}_{s}{\bf{1}}\psi \sim v{\gamma }^{0}{{\rm{\Delta }}}_{s}u$. Similarly pseudo-scalar pairing is ${\bar{\psi }}_{c}{{\rm{\Delta }}}_{5}{\gamma }^{5}\psi ={\psi }_{c}^{\dagger }{{\rm{\Delta }}}_{5}{\gamma }^{0}{\gamma }^{5}\psi \sim v{{\rm{\Delta }}}_{5}{\gamma }^{0}{\gamma }^{5}u$, etc. We have examined all of the above 16 possible superconducting pairing channels. We find that the Δ_5_ parameter gives rise to a gap closing when both *m* and Δ_5_ are present. To see this in Δ_5_ channel, let us for clarity of notation set *ħ* and *v*_*F*_ to 1 which gives the eigenvalues of Eq. (3) as $\varepsilon =\pm \sqrt{A\pm 2\sqrt{B}}$, where $A={k}^{2}+{m}^{2}+{E}_{F}^{2}+{{\rm{\Delta }}}_{5}^{2}$ and $B={{\rm{\Delta }}}_{5}^{2}{m}^{2}+({m}^{2}+{k}^{2}){E}_{F}^{2}$. When the Dirac mass *m* dominates the spectral gap (Δ_5_ → 0) the above eigenvalues reduce to $\varepsilon =\pm {E}_{F}\pm \sqrt{{k}^{2}+{m}^{2}}$. In this case each eigenvalue is doubly (spin) degenerate^27^. Deep in the 3DDS region where Δ_5_ dominates the eigenvalues acquire the following structure $\varepsilon =\pm \sqrt{{(k\pm {E}_{F})}^{2}+{{\rm{\Delta }}}_{5}^{2}}$ which corresponds to a pairing gap at the Fermi level. In a region where both *m* and Δ_5_ are non-zero, the nature of gap can be more clearly seen if we look at *E*_*F*_ = 0 where dispersion becomes $\varepsilon =\pm \sqrt{{k}^{2}+{(m\pm {{\rm{\Delta }}}_{5})}^{2}}$ and the band gap is determined by Δ_±_ = (*m* ± Δ_5_) which clearly indicates the competition between the Dirac mass *m* and the pseudo-scalar superconducting parameter Δ_5_. Figure 2 summarizes the closing and reopening of the spectral gap as one moves from *m* dominated region to Δ_5_ dominated region. The closing of the Dirac gap and re-opening of it in the form of a pseudo-scalar superconducting gap in this system does not require any magnetic field. This implies that when a Δ_5_ 3DDS is brought next to a normal 3DDM with gap parameter *m*, such that Δ_5_ > *m*, the induced Δ_5_ on the normal 3DDM side decays towards the bulk of 3DDM and crosses *m* somewhere in the interface where the excitations become gapless.

Let us now prove that when Δ_5_ = *m* a two dimensional Majorana sea appears. The pseudo-scalar character of Δ_5_ pairing can be interpreted as a spin- *singlet* superconductor whose mirror image has an opposite sign. To understand further the properties of such a pairing, let us now explicitly construct the anti-unitary operators corresponding to particle-hole, and time-reversal transformation for Eq. (3): Let *η*_*j*_ be set of Pauli matrices in the Nambu space. Then the particle-hole and time-reversal symmetries in this space can be defined as $PH=i{\eta }_{2}\otimes {\gamma }^{0}{\gamma }^{5}{\gamma }^{2}K$ and $TR={\eta }_{0}\otimes {\gamma }^{0}{\gamma }^{1}{\gamma }^{3}K$, respectively. Owing to *PH* ^2^ = 1 and *TR* ^2^ = −1, the chiral symmetry $SL=PH\ast TR$ satisfies *SL* ^2^ = 1 which places the present system in the DIII class of topological superconductors which can be classified with winding number. We explicitly obtain the topological charge^27^,

4 $${\mathscr{Q}}={\rm{\Theta }}\,(|{{\rm{\Delta }}}_{5}|-|m|)\,{\rm{sign}}\,({{\rm{\Delta }}}_{5})$$

which clearly shows that *Q* can be 0, ±1, and hence a *Z* number classification^28, 29^.

### Anomalous Andreev reflection

To further corroborate our central result concerning a sea of Majorana fermions in the present system, let us now focus on the transport properties arising from 2DMS which can be directly accessed in experiments. Let us begin by looking into a single *m*|Δ_5_ junction. The lateral coordinates in the plane of junction are (*x*, *y*) confined within a lateral dimensions (*W*_*x*_, *W*_*y*_), which means the corresponding components of wave vector in these directions are quantized as ${k}_{x(y)}=({n}_{x(y)}+\mathrm{1/2})\,\pi /{W}_{x(y)}$. Each mode can be identified with a set of quantum number *n* = (*n*_*x*_, *n*_*y*_). In the 3DDM side of the junction (*z* < 0), there are eight components of wave functions, ${{\rm{\Psi }}}_{e(h)\uparrow (\downarrow )}^{M,\pm }$ where the indices *e* (*h*) characterize electron (hole)-like quasi-particles, ↑ (↓) denotes its spin direction with respect to *z*-axis and ± indicates whether they are right- or left-movers^27^. The interface between *m* (*z* < 0) and Δ_5_ (*z* > 0) regions can reflect an incident electron as a hole. In this Andreev reflection process, the missing charge of 2*e* is absorbed by superconductor as Cooper pair^30^. Typically the s-wave nature of superconductivity imposes that the reflected hole from an ↑-spin incident electron must be in a ↓-spin state and vice versa. However, due to strong spin-orbit coupling encoded in the Dirac Hamiltonian of a 3DDM, when an electron in a given spin direction hits a 3DDM, the spin of the electron transmitted into 3DDM can be flipped^31^. Therefore in 3DDM|3DDS junction we also take into account the possibility of an anomalous –i.e. spin flipped–Andreev reflection of the reflected hole. The boundary condition for an ↑ spin electron hitting the interface is given by,

5 $$\begin{array}{l}{{\rm{\Psi }}}_{e,\uparrow }^{M,+}+\sum _{\nu =\uparrow ,\downarrow }\,{r}_{N,\alpha }{{\rm{\Psi }}}_{e,\nu }^{M,-}+\sum _{\nu =\uparrow ,\downarrow }\,{r}_{A,\nu }{{\rm{\Psi }}}_{h,\nu }^{M,-}\\ \quad \,\,\,\,=\sum _{\kappa =1,2}\,{t}_{e,\kappa }{{\rm{\Psi }}}_{e,\kappa }^{S,+}+\sum _{\kappa =1,2}\,{t}_{h,\kappa }{{\rm{\Psi }}}_{h,\kappa }^{S,+}\end{array}.$$

**Figure 3:**
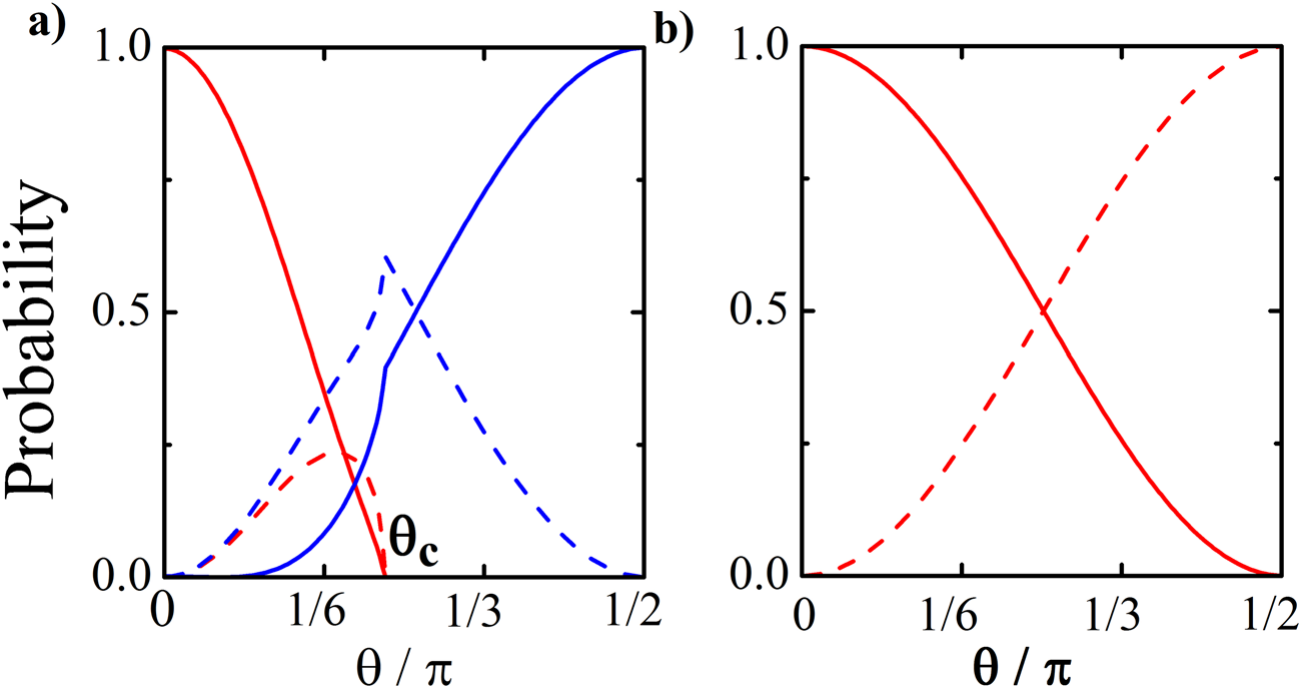
Probability of Andreev and normal reflections vs polar angle of incident. The conventional (spin-flipped) normal reflection is shown by solid (dashed) blue line. Also, the conventional (anomalous) Andreev reflection is prevaricated by solid (dashed)red line. (**a**) For *ε* = Δ_5_, the Andreev-Klein tunneling occurs in *θ* = 0. (**b**) In *ε* = 0, the perfect Andreev reflection occurs for all angles of incidence. The input values are *E*_*F*_ = *m*, Δ_5_ = 5*m*.

The left hand side is related to the wave function in 3DDM side, and the right hand side describes the wave equation in 3DDS side. Here *r*_*N*,↑_ and *r*_*N*,↓_ are the amplitudes of conventional and spin-flipped normal reflection, respectively. The *r*_*A*,↓_ and *r*_*A*,↑_ are the amplitudes of conventional and anomalous Andreev reflection, respectively. Similar processes take place for ↓ spin and hole carriers as well^27^. The probability of these reflections vs. *θ*, the polar angle of incidence, are depicted in Fig. 3. Because of the conservation of parallel component of wave vector, ${k}_{n}=\sqrt{{k}_{x}^{2}+{k}_{y}^{2}}$, at the scattering process, the angle of propagation for reflected hole (*θ*′) has a critical value ${\theta }_{C}={\sin }^{-1}\,(({E}_{F}-\varepsilon {)}^{2}-{{\rm{\Delta }}}_{D}^{2})/{({E}_{F}+\varepsilon )}^{2}-{{\rm{\Delta }}}_{D}^{2}))$ beyond which the reflected hole can not contribute to transport. For zero modes, *ε* = 0, the conventional and spin-flipped normal reflections would disappear and we are left with the conventional and anomalous Andreev reflection given by,

6 $${r}_{A,\downarrow }=-{e}^{-i\varphi }\,\cos \,\theta ,\quad {r}_{A,\uparrow }={e}^{i\alpha -i\varphi }\,\sin \,\theta ,$$

where *α* = arctan(*k*_*y*_/*k*_*x*_) is azimuthal angle. From Eq. (6), it is obvious that for a zero-energy incident electron at any angle of propagation we have a perfect Andreev reflection, ${|{r}_{A,\downarrow }|}^{2}+{|{r}_{A,\uparrow }|}^{2}=1$. This is a transport signature of Majorana fermions^9, 32^. This effect is robust against changing the chemical potential and angles of incidence (*α*, *θ*)^33^.

### Resonant peak of Majorana Zero-modes

**Figure 4:**
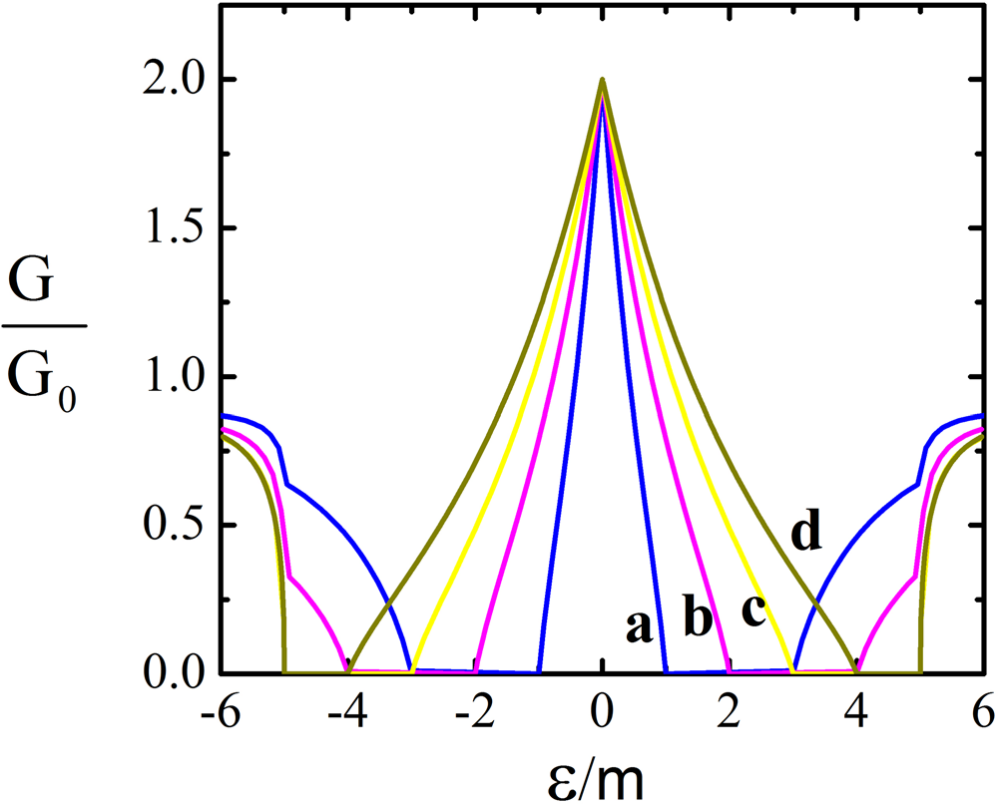
Conductance of the *m*|Δ_5_ system. We set Δ_5_ = 5*m* and *E*_*F*_/*m* = {2, 3, 4, 5} for {*a*, *b*, *c*, *d*} curves respectively.

The BTK formula for the differential conductance of the junction^34^ will be,

7 $$\frac{G(\varepsilon )}{{G}_{0}}=\sum _{n}\,[1+\sum _{\nu }\,{|{r}_{A,\nu }(\varepsilon ,{k}_{n})|}^{2}-{|{r}_{N,\nu }(\varepsilon ,{k}_{n})|}^{2}]$$

where *G*_0_ = *e* ^2^/*h* is the quantum of conductance. Note that the summation over *ν* includes spin-flipped contributions as well. When the linear dimensions of the interface are much larger than the superconducting coherence length, *ξ*_*s*_ = *ħv*_*F*_/Δ_5_, i.e. ${W}_{x},\,{W}_{y}\gg {\xi }_{s}$, the summation over mode indices *n* in Eq. (7) can be replaced by an integral. The conductance for various values of chemical potential has been plotted in Fig. 4 as a function of energy. Note that the height of the resonance peak at zero energy is pinned at 2*G*_0_ which indeed arises from the 2DMS.

### Fractional Josephson Current

**Figure 5:**
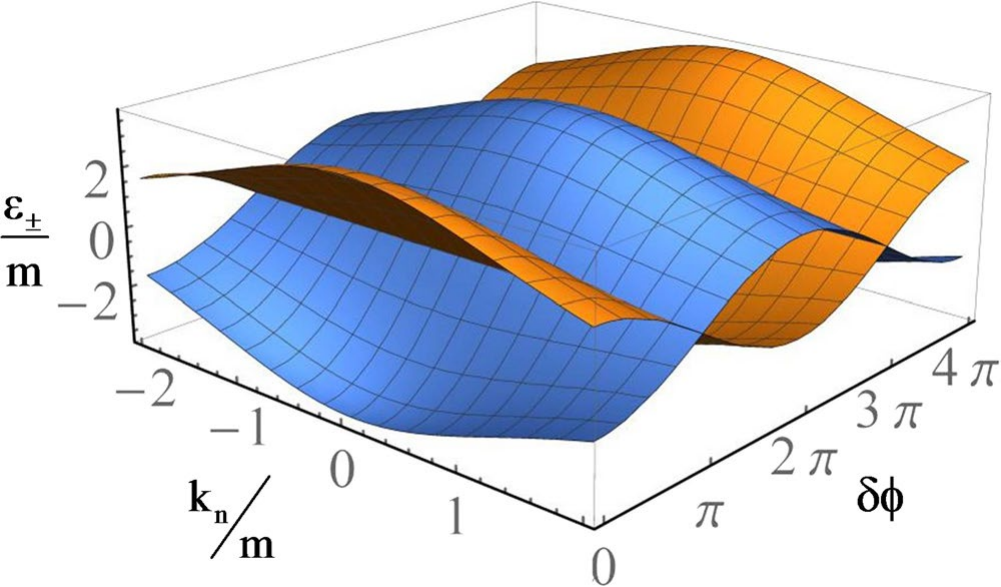
The Andreev bound states obtained for Josephson junction of part (b) in Fig. 1. These states show 4*π* periodicity with respect to *δϕ* which is an another direct signature of 2DMS.

Now let us move to the next transport signature of 2DMS, namely the fractional Josephson current. Consider the geometry depicted in part(b) of Fig. 1 and assume that *L* is the length of junction and *δϕ* = *ϕ*_*R*_ − *ϕ*_*L*_ is the phase difference between the two Δ_5_ superconductors. In th short junction limit, $L\ll {\xi }_{S}$, the Andreev bound states (ABS) responsible for carrying the super-current between two 3DDS region are given by refs 27 and 35,

8 $${\varepsilon }_{\pm ,n}(\delta \varphi )=\pm {{\rm{\Delta }}}_{S}\sqrt{{\tau }_{n}}\,\cos \,(\delta \varphi /2).$$

where *τ*_*n*_ is the normal transmission probability of the junction, ${\tau }_{n}={({\cosh }^{2}{\kappa }_{n}L+{E}_{F}^{2}{\sinh }^{2}{\kappa }_{n}L/{\kappa }_{n}^{2})}^{-1}$, with ${\kappa }_{n}=\sqrt{{m}^{2}+{k}_{n}^{2}-{E}_{F}^{2}}$. Despite that the Hamiltonian in Eq. (3) is invariant under 2*π* phase shift, *ϕ* → *ϕ* + 2*π*, the ABS in Eq. (8) clearly has a 4*π* period. This has been depicted in Fig. 5. Using Eq. (8), the corresponding Josephson current becomes, ${I}_{\pm }(\delta \varphi )=(e{{\rm{\Delta }}}_{5}/\hslash )\,{\sum }_{n}\,\partial {\varepsilon }_{\pm ,n}\,(\delta \varphi )/\partial \delta \varphi =\pm {I}_{c}\,\sin \,(\delta \varphi /2)$, where ${I}_{c}=(e{{\rm{\Delta }}}_{5}/2\hslash )\,{\sum }_{n}\,\sqrt{{\tau }_{n}}$ is the critical value of the Josephson current. In the absence of perturbations which violate fermion parity conservation, such a form of fractional Josephson current is a signature of 2DMS on the surface of 3DDS^12^.

## Discussion

**Figure 6:**
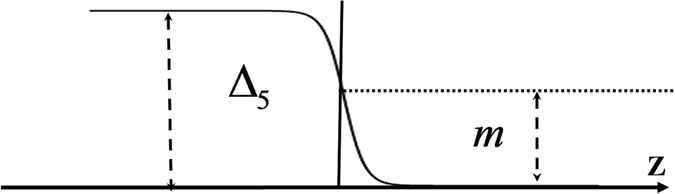
Proximity induced generation of Majorana fermions in Δ_5_|*m* setting. The right side (green) is a 3DDM which is not superconducting in the absence of a conventional superconductor (dark blue) to its left.

We have shown that a 2DMS can be obtained from singlet pairing as well, provided the superconducting order is pseudo-scalar, i.e. it changes sign under mirror reflection. Such form of superconductivity among other forms of superconductivity can be induced into the 3DDM by proximity to a conventional s-wave superconductor^25^. The two dimensional Majorana sea can emerge if we can manage to change the sign of Δ_5_ − *m*, i.e. to invert the gap parameter. If we are dealing with an intrinsic 3DDS for which Δ_5_ > *m*, its natural surface with vacuum already is where 2DMS appears, because the vacuum itself can be thought of a trivial gapped state with *m* → ∞ and Δ_5_ = 0. This leads to simpler experimental setting. However if the superconductivity is induced by proximity^25^, we only need to link a conventional superconductor to a 3DDM and the 2DMS will appear inside the 3DDM. This has been show in Fig. 6. A conventional superconductor (dark blue) to the left of a 3DDM (green) induces various forms of superconductivity into the 3DDM^25^. However, among them all, only the Δ_5_ superconductivity sets a battle against *m* to close the spectral gap. We have explicitely checked this by considering simultaneous precence of Δ_5_ and Δ_other_ where the later can be any of the 15 remaining order parameters. The surface at which the induced Δ_5_ pairing crosses the Dirac gap *m* (boundary between gray and green regions) hosts the two dimensional Majorana sea.

The present system is a singlet cousin in the family of odd-parity superconductors^20^. Our scenario for Majorana surface states does not require proximity to any magnet, as no triplet pairing is involved here. Advantages of our scenario over the existing p-wave superconductor + magnet scenarios is that

- Firt of all, it does not require p-wave superconductors which are very rare in nature.
- When contrasted to the Majorana fermions bound to a vortex core, our scenario avoids the complications associated with presence of gapless electronic excitations in the (non-superconducting Fermi liquid) vortex core itself.
- The fact that the spectral gap is a result of competition between the pseudo-scalar superconducitng pairing Δ_5_ and scalar mass *m* means that the same scenario works equally well in a 3DDM with *m*_5_ mass and a conventional (scalar) BCS superconducting order. This is simply because a suitable gauge transformation can place the minus sign reqiured in the behavior under mirror reflection on the Dirac mass rather than on the pairing term.
- Our analysis depends on the existence of *γ* ^5^ matrix which exists in odd space dimensions. Therefore by the same token Majorana zero modes in the Δ_5_|*m* or Δ_*s*_|*m*_5_ settings in one space dimension are also expected.

Perfect Andreev reflection and fractional Josephson current as two hallmarks of the ensuing Majorana sea leave clear transport footprints. The junction between such a superconductor and conventional *s* and *d* wave superconductors similar to the odd-parity p-wave superconductors may provide anomalous flux quantization in units of *h*/4*e* ^20^.

## Methods

We have used the properties of the Lorentz group to identify the pseudo-scalar nature of the Δ_5_ superconductor considered here. The characterization of the topology of the pseudo-scalar superconducting state cosidered here requires the use of topology to calculate the winding number. In evaluation of the energy spectrum, and construction of symmetry operations we have used the standard linear algebra.
